# Preclinical Trial of Traditional Plant Remedies for the Treatment of Complications of Gestational Malaria

**DOI:** 10.3390/medicines8120079

**Published:** 2021-12-17

**Authors:** Peter Uchenna Amadi, Emmanuel Nnabugwu Agomuo, Chinyere Nneka Ukaga, Uche Chinedu Njoku, Joy Adaku Amadi, Chinweuba Godswill Nwaekpe

**Affiliations:** 1 Department of Biochemistry, Imo State University, Owerri 460102, Nigeria; Nnabugwuago@gmail.com; 2 Department of Animal and Environmental Biology, Imo State University, Owerri 460102, Nigeria; chinyukaga@yahoo.com; 3 Department of Biochemistry, University of Port Harcourt, Choba 500102, Nigeria; Uche_njoku@uniport.edu.ng; 4 Department of Nutrition and Dietetics, Imo State University, Owerri 460102, Nigeria; Joyevans2020@gmail.com; 5 Department of Biochemistry, Federal University of Technology, Owerri 460102, Nigeria; Nwaekpe.chinweuba@futo.edu.ng

**Keywords:** malaria, parasitemia, pregnancy, preeclampsia, *Azadirachta indica*, *Dacryodes edulis*

## Abstract

**Background:** Most pregnant women living in high malaria endemic regions of Nigeria use herbal remedies for the management of malaria-in-pregnancy, rather than the commonly prescribed drugs. Remedies common to this area involve a suspension of *A. indica* (AI) leaves and in some cases, a suspension containing a mixture of AI and *D.* *edulis* (PS). **Aim:** This study examined the therapeutic efficacies of AI, PS, or a combination of AI and PS in a pregnant rat model for exoerythrocytic stages of *Plasmodium falciparum* parasite. **Method:** A predetermined sample size of 30 dams was used (for a power level and confidence interval of 95%), and divided equally into six groups made up of non-malarous dams, untreated malarous dams, and malarous dams either treated exclusively with 1 mL of 3000 mg/kg b.w AI, 1000 mg/kg b.w PS, AI + PS (50% *v*/*v*), or 25 mg/kg b.w CQ. **Result:** No maternal mortality was recorded. AI significantly improved maternal weight gain from 32.4 to 82.2 g and placental weight from 0.44 to 0.53 g. In the curative test, AI and AI + PS significantly reduced the average percentage parasitemia (APP) in the pregnant rats from >80% to <20%. No significant difference in the APP was found between the pregnant rats treated with any of CQ or AI during the suppressive test. Results for the prophylactic test of the study groups showed that the APP was significantly reduced from 24.69% to 3.90% when treated with AI and 3.67% when combined with PS. AI + PS reduced diastolic blood pressure from 89.0 to 81.0 mm/Hg and compared with that of the non malarous dams. AI or AI + PS significantly increased the platelet counts (10^3^ µL) from 214.1 to 364.5 and 351.2, respectively. AI and AI + PS improved birth weight from 2.5 to 3.9 g and crown rump length from 2.6 to 4.1 cm. For biomarkers of preeclampsia, combining AI and PS led to the reversal of the altered levels of creatine kinase, lactate dehydrogenase, cardiac troponin, soluble Fms-Like Tyrosine Kinase-1, and placental growth factor. **Conclusions:** This study validates the use of *A. indica* for the treatment of gestational malaria due to its antiplasmodial and related therapeutic effects and in combination with pear seeds for the management of malaria-in-pregnancy-induced preeclampsia.

## 1. Introduction

Malaria is a leading cause of deaths in low- and middle-income countries (LMICs) and a significant worldwide public health concern [1,2]. In Nigeria, malaria is a major cause of illness, maternal and fetal losses, low birth weight (LBW), and poverty, and threatens the economy and wellbeing of the nation. Approximately a $12 billion annual loss is incurred to manage the economic burden of malaria in Africa [3]. In these high endemic regions, pregnant women and children below 5 years are of particular public health interest because they constitute the category with the highest risk for malaria-related morbidity and mortality [4]. In 2018, about 11 million pregnancies in moderate and high transmission Sub-Saharan African (SSA) countries were exposed to malaria infection, with Nigeria and DRC Congo having the highest prevalence to exposure [5]. Another study reported that malaria accounts for 70% of maternal morbidity in Nigeria, 15% of maternal anemia, and 5–14% of low birth weight, with the majority of these pregnant women predicted to have two to three episodes during a particular pregnancy, four times likely to get sick from malaria, and twice likely to die from the disease [6]. Problems of implementation and effectiveness of interventions have contributed to the small margin of success achieved from these programs. In the majority of studies, malaria endemic regions are inaccessible and non-compliant with malaria action programs, while the majority of these women have no access to primary healthcare facilities, and worse still, a greater proportion of pregnant women rely on traditional medicine practitioners for ante-natal care, delivery, and postnatal care. African traditional medicine remains one of the oldest and most adopted forms of medical care delivery. The affordability, perceived efficacy, and being the most widely accepted and culturally deep-rooted tradition among Africans are primary reasons behind the high prevalence of traditional medicine use in poor-resource settings. A study on mutigravids in malaria endemic regions of Nigeria has indicated the effectiveness and suitability of herbal medicine and not due to financial constraint or lack of access to a health facility as reasons for the preference over conventional treatments [7]. Another study reported that 93% of pregnant women within 3 other malaria endemic regions of Nigeria exclusively used herbal medicine for gestational malaria, and 87.1% of these women perceived herbal medicine as effective for treating malaria [8]. It is therefore long overdue to exploit and refine these age-long treatment protocols peculiar to these areas of high malaria endemicity to support the goal of reducing the incidence of malaria-in-pregnancy in poor-resource settings. To this effect, we stratified the response of pregnant women living in areas of high malaria endemic regions of Imo State, regarding the predominant traditional practice of malaria treatment for gestational malaria, and found *A. indica* therapy had the highest prevalence. *Azadirachta indica* plants popularly known as the neem tree belong to the family of Meliaceae and have a very long-recorded wide reportage for its diverse medicinal use in the tropics, especially for malaria treatment [9,10,11] and as a potent antioxidant, anticancer, anti-inflammatory, and antidiabetic agent [12,13]. Nimbolide [9] and genudin [14] are the reported most bioactive components against *P. falciparum* and in one in vitro study, the limonoids from *A. indica* produced an IC_50_ of 0.95 pg/mL against *P. falciparum* [15]. These early studies on A. indica in addition to the evidence of use during pregnancy in real life in endemic regions of Nigeria encourages further studies. The aim of this present study was to use a pregnant rat model for the exoerythrocytic stages of the *Plasmodium falciparum* malaria parasite to verify the antimalarial effect of *A. indica* supplemented with *D. edulis* seeds as used in real life during malaria-in-pregnancy, and compared to chloroquine-treated and non-malarous controls.

## 2. Methodology

Thirty-five (35) pregnant women in Owerri North Imo State Nigeria were issued a questionnaire seeking information about traditional antimalarial methods used during gestation. Their responses were stratified and the most popular herb with a 64% prevalence taken as aqueous extract was identified as *Azadirachta indica*. Approximately 11% identified the seeds of *D. edulis* as an additional herb to control incidences of hypertension during malarous pregnancies. As justification, we initially ascertained the vasomodulatory potentials of *D. edulis* seeds [16]; hence, we decided to apply a combinatorial administration of both therapies to malarous dams.

### 2.1. Sample Collection and Preparation

Fresh leaves of *Azadirachta indica* (neem) and ripe fruits of *D. edulis* were obtained from a farmland at Imo State University, and identified by Prof. F.N. Mbagwu at the Department of Plant Science and Biotechnology, Imo State University Owerri. The leaves and fruits were thoroughly washed with distilled water to remove debris and air dried. The pear fruits were boiled and afterwards cut open to harvest seeds. Precisely 50 g of each sample were ground using a mechanical grinder and soaked in 100 mL of distilled water for 24 h. The aqueous extract of each sample was concentrated using a rotary evaporator. An acute toxicity test was carried out according to the procedure of Amadi et al. [17] to determine the LD50 of the samples on pregnant rats and was found at 1500 mg/kg for *D. edulis* and 3500 mg/kg for *A. indica*.

### 2.2. Sample Size Determination

The sample size for this study was calculated using the G*Power statistical software 3.1.9.7 with data from our pilot study measuring blood schizonticidal activity as the primary outcome. The pilot study compared the blood schizonticidal activity of *A. indica*, *D. edulis*, and both mixtures on malarous rats with malarous rats treated with chloroquine. The standard deviation within each group (σ), otherwise known as the combined standard deviation, was estimated at 34.0415%. With these details, the effect size (f) was automatically generated as 0.9754114 with the G*Power statistical software. We decided to be maximally idealistic and chose α = β = 0.05, implying a power level of 1 − β = 0.95 (95%) and at a 95% significance level, a sample size of 25 experimental rats was estimated using the statistical tool and with a similar group size for non-malarous dams, a total of 30 pregnant (30) rats were required for this study.

### 2.3. Animal Handling

The animal handling completely aligned with the Principles of Laboratory Animal Care (NIH Publication No. 85-23). Ethical permit for this study was issued by the Biochemistry Research and Ethics Board, Imo State University (IMSU/BCM/ETS/20181212). Precisely 20-week-old inbred adult non-pregnant female rats, and 16-week-old adult rats were used for this study. The weights of male rats were from 150–180 g while female rats were from 190–220 g. Vaginal lavage was used for the determination of the female estrous cycle in the rats and afterwards, the rats were separated and housed overnight in different metabolic cages of 3 female rats to 1 male rat at the time of mating. Normal rat feed pellets (UAC Nigeria Grand Cereals Vital Feeds, Jos, Plateau State, Nigeria) and water *ad libitum* were provided. The female rats were tested daily for copulation and a positive vaginal smear check/mating plug confirmed pregnancy and served as the first day of gestation (G0). Thirty pregnant rats were selected and housed individually in metabolic cages with suitable bedding.

### 2.4. Inoculation of Pregnant Rats

Inoculums consisting of 5 × 10^7^ infected erythrocytes were obtained from donor rats previously infected with *Plasmodium falciparum* strains NF54. The *P. falciparum* strain was rat-adapted from a mouse strain by three successive 4-week passages through 7-week-old rats [18]. Parasitemia of the donor’s blood was first determined at 81%. The inoculums were diluted with 0.9% physiological saline and red blood cells of uninfected rats to obtain 1 × 10^7^ *P. falciparum*-infected erythrocytes. Twenty-five pregnant rats were inoculated intravenously with 0.2 mL inoculums of the diluted infected erythrocytes and confirmed 3 h later by microscopy using the tail blood of the rats. The day of inoculation was defined as day zero (D0).

### 2.5. Grouping, Feeding, and Sample Collection

The experimental animals were divided into 6 study groups: group 1 was the negative control (-ve ctrl) representing the untreated malarous dams, group 2 (inf + CQ) was the malarous dams treated with 25 mg/kg chloroquine, group 3 (inf + AI) was the malarous dams treated with 3000 mg/kg b.w *A. indica*, group 4 (inf + PS) was the malarous dams treated with 1000 mg/kg b.w pear seed extracts, group 5 (inf + AI + PS) was the malarous dams treated with 50% *v/v* of 3000 mg/kg b.w *A. indica* and 1000 mg/kg b.w pear seeds, and group 6 (baseline) was the non-malarous dams. All treatments were made by oral gavaging with 1 mL of the test compounds administered once daily. All rats had free access to normal rat chow and water.

Treatment lasted for 21–23 days until parturition.

### 2.6. Measurement of Maternal Hemodynamics, Pregnancy Outcomes, and Sample Collection

Maternal weight before copulation and prior to delivery was recorded using sensitive digital weighing balance, as was the feed intake. Urine output was collected on gestation day 19, and measured with a Thermo Fisher Scientific, Inc. (Waltham, MA, USA). Pierce™ BCA protein assay kit [19]. The pups were counted according to live and still born, weighed using the digital weighing balance, measured for crown rump length with Vernier calipers, and monitored for time taken for eye opening, and appearance of furs.

The blood pressure and heartbeat rate were measured using the tail cuff-based methods with a CODA non-invasive blood pressure (NIBP) system (Kent Scientific Corporation, Torrington, CT, USA). The platelet count was determined with the BC-2600 model of haematology autoanalyzer (Shenzhen Mindray Bio-medical Electronics, Shenzhen, China) according to the user manual.

Maternal blood (3 mL) was collected with a 5 mL disposable syringe immediately after delivery by cardiac puncture. The rats were first anesthetized with 3% intraperitoneal pentobarbital sodium (50 mg/kg). Animals were then sacrificed via exsanguination, and an autopsy was carried out to remove and weigh the placenta using a sensitive digital weighing balance. Serum was prepared by centrifugation of the blood samples at 4000 rpm for 10 min, transferred into a tube, and freeze-stored at 20 °C pending analysis. 

### 2.7. Determination of Antimalarial Activity 

For the antimalarial assay, a separate experimental set up involving 40 pregnant rats equally divided across the four test groups were used to evaluate the prophylactic and suppressive potentials of the chloroquine, exclusive *A. indica*, and *D. edulis*, administration, and a combined *A. indica* and *D. edulis* treatment.

The Rane’s test was performed according to the method described by Ryley and Peters, [20] to determine the curative effects of the test compounds. The chemosuppressive potentials of the test compounds against the *P. falciparum* infectio were determined using the Peter’s four-day suppressive test [21] while the prophylactive effect of the test compounds was determined according to the description of Peters [22]. The rectal temperature was measured using a digital rectal thermometer. The average percentage parasitemia, and suppression were calculated as follows [23]:

$$\%\;\mathrm{Parasitemia}=\frac{\;\mathrm{Number}\;\mathrm{of}\;\mathrm{parasitized}\;\mathrm{RBC}}{\mathrm{Total}\;\mathrm{number}\;\mathrm{of}\;\mathrm{RBC}\;\mathrm{count}}\times 100\;\%\;\mathrm{Suppression}=\frac{\%\mathrm{Parasitemia}\;\mathrm{in}\;\mathrm{negative}\;\mathrm{control}\;-\%\mathrm{Parasitemia}\;\mathrm{in}\;\mathrm{study}\;\mathrm{group}}{\%\mathrm{Parasitemia}\;\mathrm{in}\;\mathrm{negative}\;\mathrm{control}}\;\times \;100$$

### 2.8. Clinical Chemistry

An enzymatic method according to the procedures in the Sigma-Aldrich assay kits were used for the determination of total cholesterol (TC), triglycerides (TGs), and high density lipoprotein cholesterol (HDL) with a BT-3000 auto analyzer (Biotecnica Instruments, Licenza, 18-00156 Rome Italy). Low-density lipoprotein (LDL), cardiac risk ratio (CRR), and atherogenic coefficients (ACs) were calculated as follows [24]:

LDL = TC − HDL − TG/5, CRR = TC/HDL, AC = CRR − 1

Creatine kinase was assayed by spectrophotometry at an absorbance of 450 nm according to the assay protocol described by Horder et al. [25]. Lactate dehydrogenase was determined by enzymatic colorimetry using the methods described in the Lactate Dehydrogenase Kit (Sigma Aldrich St. Louis, MO, USA, Catalog No-MAK066) while the cardiac troponin test was performed by enzyme-linked immunosorbent assay (ELISA) in accordance with the procedures on the ELISA kit (East Biopharm, Hangzhou, Zhejiang, China). Soluble Fms-Like Tyrosine Kinase-1 (sFlt-1) and placental growth factor (PIGF) was determined by quantitative sandwich enzyme immunoassays using commercial ELISA kits from Mybiosource San Diego, CA, USA.

### 2.9. Statistical Analysis

The data obtained was analyzed and presented as the means ± standard deviations of five determinations. A test of normality was performed on the data obtained before analysis using the Shapiro Wilk’s Test to confirm the normality of the distribution of the values. An SPSS version 20 statistical tool was used for the computation of the means and standard deviations, and a test of significance at 95% confidence by One-Way Analysis of Variance (ANOVA) among the test groups. The degree of correlation between the Average Percentage Parasitemia (curative) and biomarkers of myocardial infarction and preeclampsia was analyzed using the Pearson’s correlation function of the GraphPad Prism software version 7.04 (GraphPad Software, San Diego, CA, USA). The parameters represented graphically were performed with the GraphPad Prism software version 7.04.

## 3. Results

Table 1 shows the morphometric features of the malarous dams in the different study groups. No maternal mortality was recorded across the study groups. Without treatment, the feed intake of the malarous pregnant rats significantly reduced from 756.4 to 498.6 g. When treated with chloroquine (CQ), a significant increase in feed intake up to 680 g was observed when compared to the untreated malarous dams while the malarous dams treated with *Azadirachta indica* (AI) significantly increased to 701.8 g compared with feed intake of the CQ-treated dams. The feed intake of the malarous dams treated with a combination of AI and pear seeds (PSs) was not significantly different from those of the non-malarous dams. The weight gain and placenta weight of the untreated malarous pregnancies were significantly lower than the treatment groups and baseline. Malarous dams treated with AI or combined with PS significantly increased the weights of the malarous dams compared to the baseline. PS administration increased the weights of the malarous rats comparably to the non malarous rats. The placenta weight of the untreated malarous dams was significantly lower than the non-malarous dams and the CQ-treated malarous dams. No significant difference was found between the placenta weight of the treatment groups and the baseline.

**Figure 1:**
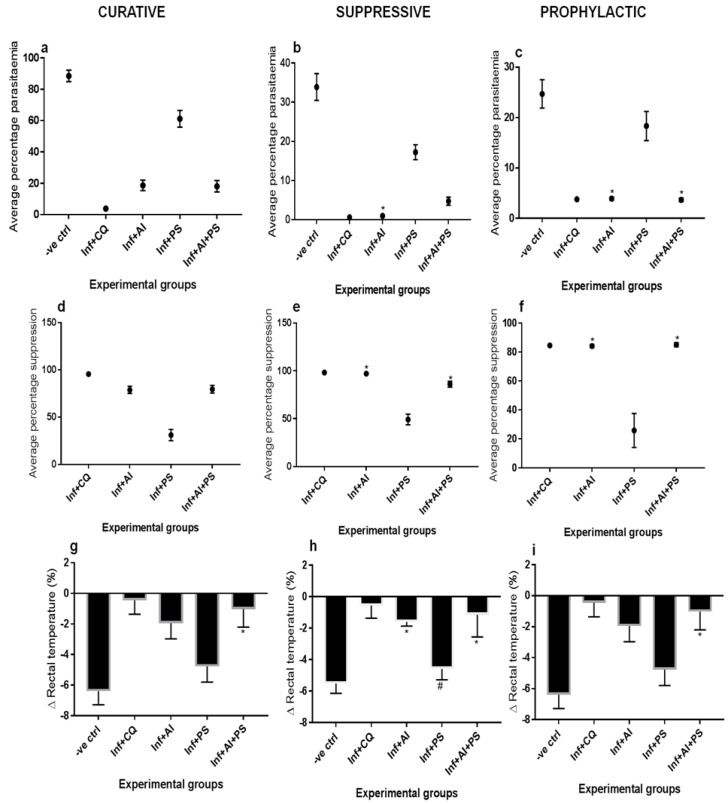
Average percentage parasitemia; curative (**a**), suppressive (**b**), and prophylactic (**c**), average percentage suppression; curative (**d**), suppressive (**e**), and prophylactic (**f**), percentage Δ in rectal temperature; curative (**g**), suppressive (**h**), and prophylactic (**i**). -ve ctrl—malarous dams untreated, inf + CQ—malarous dams treated with chloroquine, inf + AI—malarous dams treated with *A. indica*, inf + PS—malarous dams treated with pear seeds, inf + AI + PS—malarous dams treated with *A. indica* and pear seeds. *—no significant difference (*p* < 0.05) with the chloroquine-treated malarous dams, #—no significant difference with untreated malarous dams.

Figure 1 shows the blood schizontocidal activities of *A. indica* (AI), pear seeds (PSs), and when combined, as compared to the effect of chloroquine (CQ) in the malarous dams. During the curative test, AI and when combined with PS significantly reduced the average percentage parasitemia (APP) in the pregnant rats from >80% to <20%. The suppressive test showed a significantly reduced APP of up to 0.99% when the malarous dams were treated with AI or 4.7% when AI was combined with PS. No significant difference in the APP was found between the pregnant rats treated with CQ or AI. Results for the prophylactic test of the study groups showed that the APP was significantly reduced from 24.69% to 3.90% when treated with AI and 3.67% when combined with PS. In comparison with the CQ-treated malarous dams, no significant difference was found with the AI and AI + PS-treated malarous dams. The exclusive treatment with PS produced 61% APP during the Rane’s test, and 17.2% and 18.3%, respectively, with the suppressive and prophylactic tests. AI, PS, and AI + PS respectively produced up to 95.60%, 78.86%, 30.89%, and 79.49% average percentage suppression (APS) against 95.60% APS for CQ with the curative test, while each of AI and AI + PS produced >85% APS in the suppressive test against 98% APS for CQ, and 84.17% and 85.12% APS, respectively, during the prophylactic test, against 84.63% APS for CQ. PS-exclusive treatment achieved 49.10% and 25% APS, respectively, during the suppressive and prophylactic tests. Any of the AI or AI + PS treatments produced no significant change in the APS when compared to the CQ effect during the suppressive and prophylactic tests. The percentage decrease in the rectal temperature (RT) of the untreated malarous dams during the curative, suppressive, and prophylactic tests was 7.5%, 5.5%, and 6.38%, respectively. Across the three tests, CQ treatment significantly decreased the RT by ≤0.5%; the AI treatment significantly decreased the RT by 1.72%, 1.60%, and 1.95%, respectively; while the AI + PS significantly decreased the RT by ≤1.1% and showed no significant difference with the CQ-treated group. The RT of the PS-treated group during the suppressive test remained similar to the untreated malarous dams.

The hemodynamics characteristics of the malarous and non-malarous dams are shown in Table 2. The untreated malarous dams showed significantly elevated systolic and diastolic BP, heartbeat rate, and urine protein elevation when compared with their respective baselines. The systolic and diastolic blood pressures of the treatment groups were comparable to the baseline. The heartbeat rates of the malarous dams treated with either exclusive PS or in combination with AI were comparable to the levels found for both the CQ-treated group and baseline. No significant difference was found between the urine protein excretion levels of the malarous dams treated with CQ and AI, while the group treated exclusively with PS showed comparable results to the baseline. Treatment with AI and in combination with PS showed that the platelet counts of both groups were significantly higher than the CQ-treated subjects and comparable to the baseline levels.

In Table 3, the untreated malarous dams produced an average of 9 pups, where 50% of these pups were stillborn with a significantly lower crown rump length and birth weight compared to the baseline and treated malarous dams. All the malarous dams treated with either AI, PS, or the combination showed a comparable total number of pups with the non-infected rats (12 pups), while only the infected rats treated with AI produced an equivalent number of still born rats (1 pup) compared to the CQ-treated rats. Infected rats treated with AI and when combined with PS produced pups with higher birth weights than CQ-treated rats but were statistically comparable to the baseline values. No significant differences were found in the time taken for fur appearance in the pups across the study groups. The pups delivered by the infected rats treated with AI and AI + PS recorded a significantly greater crown rump length than both the CQ-treated dams and the non-infected rats.

The lipid profile of pregnant rats infected with the *P. falciparum* parasite and either left untreated or treated with CQ, AI, PS, or AI + PS is shown in Table 4. The total cholesterol (TC) and triglyceride (TG) and low-density lipoprotein (LDL) levels of the malarous dams were significantly higher than those of the non malarous dams. The TC, TG, and LDL levels of malarous dams treated with PS or when combined with AI were comparable to the baseline, while the malarous dams treated with either CQ or AI showed significantly higher TC and TG compared to the baseline levels. No significant differences were observed between the individual HDL levels of the treatment groups and baseline. The cardiac risk ratio (CRR) and atherogenic coefficient (AC) of the untreated malarous dams were significantly higher than the non-malarous dams. Chloroquine slightly lowered the CRR levels of the malarous dams when compared to the untreated dams but were significantly higher than the baseline levels. The malarous dams treated with AI, PS, and AI + PS showed comparable CRR and AC levels with the baseline values.

**Figure 2:**
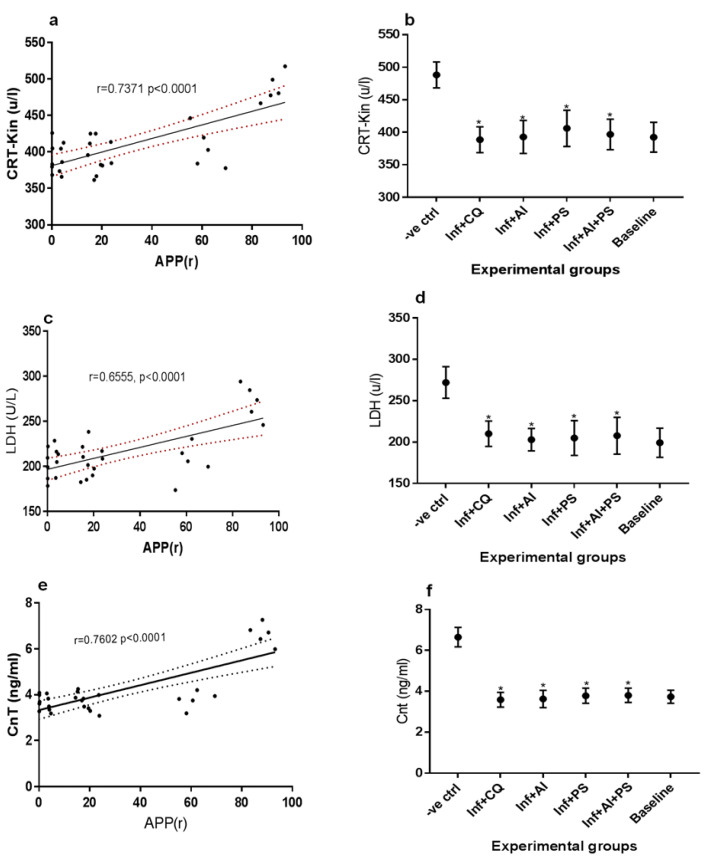
(**a**) Correlation between Average Percentage Parasitemia Rane’s (APP(r)) vs. creatinine kinase (Crt-Kin), (**b**) mean creatinine kinase levels of malarous and non malarous dams in the study groups, (**c**) Correlation between Average Percentage Parasitemia Rane’s (APP(r)) vs. lactate dehydrogenase (LDH), (**d**) mean lactate dehydrogenase levels of malarous and non malarous dams in the study groups, (**e**) Correlation between Average Percentage Parasitemia Rane’s (APP(r)) vs. cardiac troponin (CnT), (**f**) mean cardiac troponin levels of malarous and non malarous dams in the study groups. -ve ctrl—malarous dams untreated, inf + CQ—malarous dams treated with chloroquine, inf + AI—malarous dams treated with *A. indica*, inf + PS—malarous dams treated with pear seeds, inf + AI + PS—malarous dams treated with *A. indica* and pear seeds, baseline—non malarous rats, *—no significant difference (*p* < 0.05) with the non malarous dams.

The correlations between the average percentage parasitemia and creatinine kinase, lactate dehydrogenase, and cardiac troponin are demonstrated in Figure 2a, Figure 2c, and Figure 2e, respectively, while the mean creatinine kinase, lactate dehydrogenase, and cardiac troponin levels of the experimental animals are shown in Figure 2b, Figure 2d, and Figure 2f, respectively. A statistically significant strong positive correlation was found between the average percentage parasitemia and creatinine kinase (*p* < 0.0001, r = 0.7371), lactate dehydrogenase (*p* < 0.0001, r = 0.6555), and cardiac troponin (*p* < 0.0001, r = 0.7602) of the test groups. The result in Figure 2b shows that the creatinine kinase levels of the untreated malarous dams were significantly elevated during the late trimester. Treatment of the malarous dams using any of CQ, AI, PS, and AI + PS produced equivalent creatinine kinase, lactate dehydrogenase, and cardiac troponin levels when compared with the baseline.

**Figure 3:**
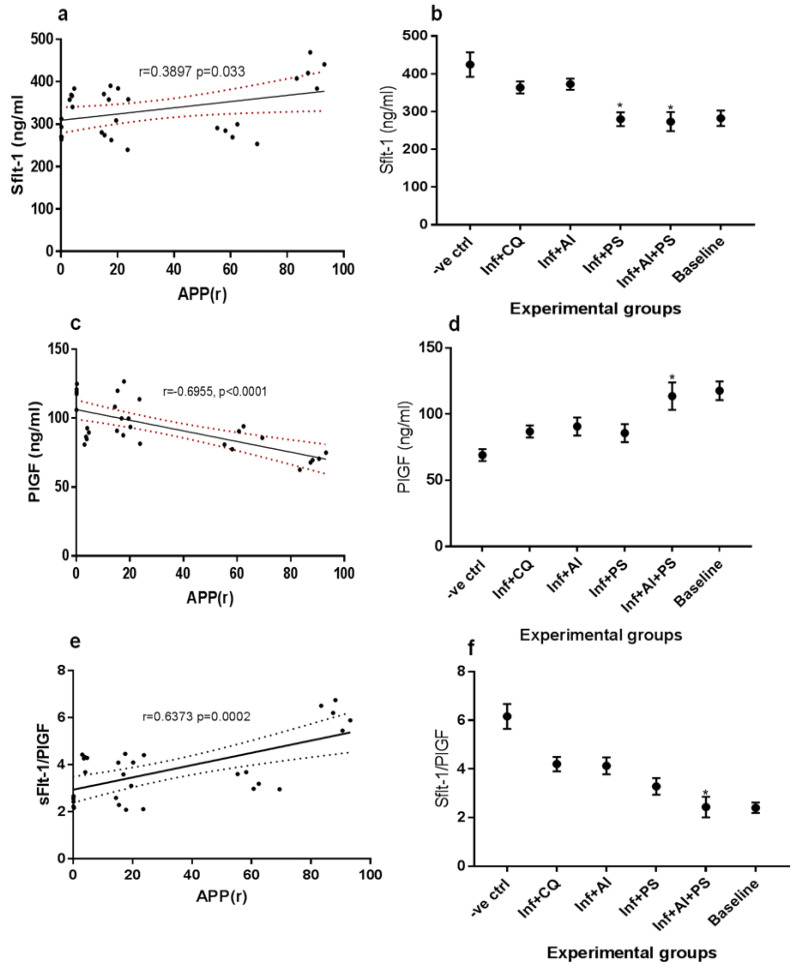
(**a**) Correlation between Average Percentage Parasitemia Rane’s (APP(r)) vs. soluble Fms-Like Tyrosine Kinase-1 (sFlt-1), (**b**) mean sFlt-1 levels of malarous and non malarous dams in the study groups, (**c**) Correlation between Average Percentage Parasitemia Rane’s (APP(r)) vs. Placental growth factor (PIGF), (**d**) mean PIGF levels of malarous and non malarous dams in the study groups, (**e**) Correlation between Average Percentage Parasitemia Rane’s (APP(r)) vs. ratio of sFlt-1 and PIGF, (**f**) mean sFlt-1/PIGF levels of malarous and non malarous dams in the study groups. -ve ctrl—malarous dams untreated, inf + CQ—malarous dams treated with chloroquine, inf + AI—malarous dams treated with *A. indica*, inf + PS—malarous dams treated with pear seeds, inf + AI + PS—malarous dams treated with *A. indica* and pear seeds, baseline—non malarous rats, *—no significant difference (*p* < 0.05) with the baseline.

The markers of preeclampsia were examined at the third trimester in the malarous dams and are reported in Figure 3a–f. The correlation between the average percentage parasitemia and soluble Fms-Like Tyrosine Kinase-1 (sFlt-1) is shown Figure 3a, while the mean sFlt-1 levels of the experimental animals are shown in Figure 3b. A statistically significant (*p* < 0.0001) positive correlation (r = 0.033) was found between the average percentage parasitemia and sFlt-1 levels. The mean sFlt-1 levels of the untreated malarous dams were significantly elevated prior to delivery while the malarous dams treated with bot AI and CQ showed slightly decreased sFlt-1 levels but were not comparable to the baseline levels. The malarous dams treated with PS or in combination with AI showed no significant difference in their sFlt-1 levels when compared with the non-malarous dams. Figure 3c showed a statistically significant (*p* < 0.0001) strong negative correlation (r = −0.6955) between the average percentage parasitemia and placental growth factor (PIGF). The result for the mean PIGF levels of the experimental animals presented in Figure 3d showed that the untreated malarous dams recorded significantly decreased PIGF when compared to the non-malarous dams. The malarous dams treated with CQ, AI, and PS significantly increased the PIGF levels but were not comparable to the baseline levels, while the malarous dams treated with a combination of AI and PS showed no significant difference in the PIGF levels compared to the baseline. A strong positive correlation (r = 0.6373) was observed between the average percentage parasitemia and ratio of sFlt-1 and PIGF (Figure 3e). The untreated malarous dams showed significantly elevated mean sFlt-1/PIGF levels while the malarous dams treated with CQ, AI, or PS showed significantly lower mean sFlt-1/PIGF levels when compared to the untreated malaria rats. The malarous dams placed on combinatorial administration of AI and PS showed mean sFlt-1/PIGF levels equivalent to the baseline levels.

## 4. Discussion

The present study was conducted on *P. falciparum*-parasitized rat models to evaluate the potency and maternal effects of a popular antimalarial herb (*Azadirachta indica* combined with *D. edulis* seeds) used in real life in areas of high malaria endemicity in Nigeria. Preliminary findings revealed that the feed intake, maternal weight, and placenta weight at birth were all adversely affected by malaria-in-pregnancy. Appetite loss was restored in rats treated with AI and in combination with PS. According to Ndyomugyenyi et al. [26], loss of appetite is one of the major diagnostic symptoms of malaria, which in this study explains the significant decrease in the feed intake and consequent weight loss of the untreated malarous dams. Maternal undernutrition is a serious risk factor for fetal undernutrition that causes changes in fetal structure and physiology and consequently, low birth weight [27]. *A. indica*, similar to the effects of chloroquine, improved the feed intake of the malarous pregnant rats, which led to the improvement of maternal weight during malaria-in-pregnancy. In agreement with the findings of this study, earlier reports associated weight gain with the administration of *A. indica* [28]; however, there is scarce literature on the effect of AI on appetite and feed intake during gestational malaria. Our study further showed that *P. falciparum* infection caused a significant decrease in placental weight. The placental weight at birth represents an important clinical parameter that determines pregnancy outcomes [29]. Several complications of maternal health are associated with low placental weight, including chronic hypertension and preeclampsia [30], and anemia [31]. Thus, the restoration of placental weight after treatment with *A. indica* is indicative of a possible efficacy in malaria-induced preeclamptic conditions. Further, the *A. indica* leaves, when administered as a prophylactic and suppressive agent against *P. falciparum* in the pregnant rats, achieved a therapeutic efficacy of 97% but slightly lesser when combined with pear seeds. This puts the potency of *A. indica* above the 33 African antimalarial herbs screened for their antiplasmodial effects [32] and within the 93% recommendations of World Health Organization (WHO) for cure rates of antimalarial drugs. Results obtained from the rectal thermometry also validated both the blood schizontocidal activities of *A. indica* especially in combination with pear seeds, as well as their potent antipyretic effect, having showed a hypothermic effect on the malarous dams. Additionally, the malarous dams compared to the non malarous dams showed symptoms of preeclampsia with an elevation of blood pressure, heartbeat rate, and urine excretion. The exclusive treatment with *A. indica* normalized both the systolic and diastolic blood pressure but showed slight efficacy in reversing an altered heartbeat and polyuria. From the findings, the practice of combining *A. indica* and pear seeds by traditional healers proves more effective in ameliorating the altered markers of malaria-induced hypertension, both of which are established symptoms of gestational malaria [33,34]. The malarous dams also showed symptoms of thrombocytopenia, which has become an established symptom and a considered prognostic factor of severe gestational malaria [35,36,37]. The administration of *A. indica* and in combination with *D. edulis* seeds led to about a 47% increase in the platelet count during the malarous pregnancy. This may be due to the reported thrombocytosis associated with the administration of *A. indica* [38]. Results from the litter characteristics further indicated that *A. indica* could complement present malaria control programs in averting poor pregnancy outcomes, fetal mortality, and low birth weight associated with malaria-in-pregnancy. The infected but untreated rats recorded about 50% fetal wastages when compared to the rats treated with *A. indica*, pear seeds, or the combination. The treatment with exclusive *A. indica* produced the least number of still born pups compared to other interventions and compared to non-malarous pregnancies. The herbs administered prevented the occurrence of low birth weight in the malarous dams, which were significantly higher than chloroquine-treated rats. Birth weight is an important primary endpoint in measuring the success of interventions during gestational malaria, with low birth weight an outcome of unsuccessful interventions. With this, *A. indica* could be considered for its overall improvement of pregnancy outcomes during gestational malaria. Additionally, from the crown rump lengths of the fetus, the interventions possibly averted malaria-induced intrauterine growth restriction. The untreated infected dams produced fetuses with significantly lower crown rump length by up to 0.6 cm, which depicted severe fetal growth restriction during gestational malaria. This result raises the suspicion that the bioactive components of the test herbs permeate the placental-fetal membrane barrier to enhance fetal well-being. Further cardiovascular involvements during malaria-in-pregnancy were highlighted by our findings. The lipid profile of the malarous dams implied that severe hypercholesterolemia was a consequence of untreated gestational malaria and the hypercholesterolemic effect persisted even after chloroquine treatment. In agreement with our finding, earlier studies established that patients with malaria present clinical conditions, such as hypocholesterolemia, hypertriglyceridemia, and low levels of high-density lipoproteins (HDLs) and low-density lipoproteins (LDLs) [39]. Hence, the therapeutic efficacy of the antimalarial herbs decreased the susceptibility of the malarous pregnant rats’ to cardiovascular dysfunction. In support of these findings, creatine kinase and lactate dehydrogenase and cardiac troponin, which are all established biomarkers of preeclampsia [40,41,42], were all significantly elevated in untreated gestational malaria, whereas they were significantly lowered to baseline levels in all the treatment groups. These elevated peptides affirm a previous suspicion that malaria-in-pregnancy predisposes to hypertensive disorders especially during the late trimester. Due to the established specificity of these peptides for cardiac tissue, it is safe to assume that some degree of myofibrillary damage occurred in association with malaria-in-pregnancy. These claims of preeclampsia resulting from gestational malaria in this study are supported by the strong correlation between the percentage parasitemia and other secondary outcome measurements like the soluble Fms-Like Tyrosine Kinase-1 (sFlt-1) and placental-induced growth factor (PIGF). sFlt-1 is a placenta-derived antiangiogenic protein regarded as a reliable diagnostic marker for preeclampsia [43]. The sFLT-1 was over-secreted during the late-stage trimester in the malarous dams and neither chloroquine nor *A. indica* exclusively administered reversed this outcome. However, pear seed either exclusively administered or in combination with *A. indica* normalized the altered sFlt-1 in the malarous dams. Hence, *D. edulis* pear seed, similar to its vasoprotective effects [16], could be regarded as a potent herb for the management of malaria-induced preeclampsia. This clearly justifies, pending clinical trials, the rationale behind the inclusion of pear seeds as part of the traditional treatment protocol for malaria in poor-resource settings. Similar to sFlt-1, PlGF is a key molecule in angiogenesis and vasculogenesis during embryogenesis with a reliable predictive performance during preeclampsia diagnosis [44]. Furthermore, the ratio of sFLT-1 and PIGF is regarded as a more economic and reliable indicator for preeclampsia and severity rather than individual interpretations [45,46,47]. Hence, the reversal of altered levels of sFlt-1/PlGF clearly supports a combinatorial administration as most effective in the management of malaria-in-pregnancy-induced preeclampsia. From the findings of this study, it is possible to hypothesize that the exclusive administration of the antimalarial drugs is less effective in mitigating the malaria-induced preeclampsia and does require supplementation with vasomodulatory agents for effective treatment of gestational malaria-induced hypertensive disorders. However, an important limitation to acknowledge in this study is the lack of data on the effect of this therapy on placenta oxidation markers. We recommend that future studies should examine if these herbal remedies modulate placenta oxidation during preeclampsia to add further credibility to the efficacy of these therapies to ameliorate malaria-induced preeclampsia.

## 5. Conclusions

The popular practice of using *A. indica*, and in some cases combining it with antihypertensive herbs like pear seeds, during malaria-in-pregnancy may be justified given the findings of this study. Early indicators of pregnancy outcomes including maternal and placental weight were effectively restored after alterations in the *P. falciparum*-infected dams. The percentage parasitemia obtained with treatment with *A. indica* was comparable to the standard drugs and attained the 93% recommended cure rates by the World Health Organization. In addition, elevated blood pressure, heartbeat rates, and thrombocytopenia found in untreated malaria-in-pregnancy were reversed with combinatorial administration of *A. indica* and the pear seeds. The use of *A. indica* as antimalarial therapy minimized fetal deaths and showed similar birth weights to non-malarous dams, and by combining *A. indica* with pear seeds, the resulting biomarkers of preeclampsia and cardiovascular dysfunction in the malarous dams were effectively normalized.

## Figures and Tables

**Table 1:** Maternal characteristics of malarous dams in the different study groups.

Groups	Maternal Mortality (%)	Feed Intake (g)	Weight Gain (g)	Placenta Weight (g)
-ve ctrl	0	498.6 ± 18.7	32.4 ± 4.3	0.44 ± 0.02
Inf + CQ	0	680.0 ± 26.2	75.6 ± 4.8	0.53 ± 0.01
Inf + AI	0	701.8 ± 22.0 ^a^	82.2 ± 4.1	0.53 ± 0.02 ^c^
Inf + PS	0	632.6 ± 30.8	71.6 ± 3.2 ^c^	0.53 ± 0.02 ^c^
Inf + AI + PS	0	733.8 ± 28.3 ^b^	88.0 ± 2.6	0.54 ± 0.03 ^c^
Baseline	0	756.4 ± 15.1	76.8 ± 5.9	0.54 ± 0.03

-ve ctrl—malarous dams untreated, inf + CQ—malarous dams treated with chloroquine, inf + AI—malarous dams treated with *A. indica*, inf + PS—malarous dams treated with pear seeds, inf + AI + PS—malarous dams treated with *A. indica* and pear seeds, baseline—non malarous rats, ^a^—no significant difference (*p* < 0.05) with the chloroquine-treated malarous dams, ^b^—no significant difference with the non malarous dams, ^c^—no significant difference with both the chloroquine-treated malarous dams and non malarous dams.

**Table 2:** Hemodynamics properties of malarous and non malarous dams.

Groups	Systolic BP (mm/Hg)	Diastolic BP (mm/Hg)	HBR (Beats/min)	24 h Urine Protein Excretion (mg)	Platelets Counts (× 10^3^ µL)
-ve ctrl	136.0 ± 4.1	89.0 ± 2.2	405.8 ± 17.8	0.83 ± 0.03	214.1 ± 19.3
Inf + CQ	122.0 ± 2.7	84.0 ± 4.1	332.0 ± 8.6	0.79 ± 0.01	314.6 ± 20.4
Inf + AI	123.0 ± 2.7 ^c^	83.0 ± 2.7 ^c^	366.4 ± 6.8	0.77 ± 0.01 ^a^	364.5 ± 14.9 ^b^
Inf + PS	123.0 ± 2.7 ^c^	80.0 ± 0.0 ^c^	319.6 ± 5.7 ^c^	0.62 ± 0.05 ^b^	249.9 ± 17.0
Inf + AI + PS	122.0 ± 2.7 ^c^	81.0 ± 2.2 ^c^	332.0 ± 10.6 ^c^	0.67 ± 0.02	351.2 ± 19.1 ^b^
Baseline	122.0 ± 2.7	78.0 ± 2.7	321.6 ± 6.0	0.59 ± 0.02	356.6 ± 26.9

-ve ctrl—malarous dams untreated, inf + CQ—malarous dams treated with chloroquine, inf + AI—malarous dams treated with *A. indica*, inf + PS—malarous dams treated with pear seeds, inf + AI + PS—malarous dams treated with *A. indica* and pear seeds, baseline—non malarous rats, ^a^—no significant difference (*p* < 0.05) with the chloroquine-treated malarous dams, ^b^—no significant difference with the non malarous dams, ^c^—no significant difference with both the chloroquine-treated malarous dams and non malarous dams.

**Table 3:** Litter characteristics of pups from malarous dams in the study groups.

Groups	Total No. of Pups	No. of Live Pups	No. of Still Born	Av. Pup Weight (g)	Eye Opening (Days)	Appearance of Fur (Days)	Crown Rump Length (cm)
Baseline	12.0	11.0	1.0	3.6 ± 0.2	15.0 ± 1.0	5.6 ± 0.8	3.3 ± 0.1
-ve ctrl	9.2 ± 1.4 ^b^	4.6 ± 1.1 ^b^	4.6 ± 0.8 ^b^	2.5 ± 0.2 ^b^	15.0 ± 0.7 ^a^	5.6 ± 0.8	2.6 ± 0.1 ^b^
Inf + CQ	12.4 ± 1.1	11.4 ± 1.1	1.0 ± 0.2	3.1 ± 0.2	15.0 ± 1.0	6.0 ± 0.7	3.3 ± 0.1 ^b^
Inf + AI	12.0 ± 1.8 ^a^	10.8 ± 1.3 ^a^	1.2 ± 0.2 ^a^	3.9 ± 0.3 ^c^	15.0 ± 0.7 ^a^	5.4 ± 0.5	4.1 ± 0.2
Inf + PS	11.8 ± 0.8 ^a^	9.4 ± 1.6 ^a^	2.4 ± 0.4	2.8 ± 0.1 ^a^	15.2 ± 0.8 ^a^	5.6 ± 0.8	3.2 ± 0.2 ^a^
Inf + AI + PS	12.6 ± 1.1 ^a^	10.6 ± 1.6 ^a^	2.0 ± 0.5	3.9 ± 0.1 ^c^	14.8 ± 0.8 ^a^	5.8 ± 0.8	4.1 ± 0.2

-ve ctrl—malarous dams untreated, inf + CQ—malarous dams treated with chloroquine, inf + AI—malarous dams treated with *A. indica*, inf + PS—malarous dams treated with pear seeds, inf + AI + PS—malarous dams treated with *A. indica* and pear seeds, ^a^—no significant difference (*p* < 0.05) with the chloroquine-treated malarous dams, ^b^—Significantly lower than baseline, ^c^—significantly higher than the CQ-treated group but comparable to baseline.

**Table 4:** Lipid profile of malarous and non malarous dams in the study groups.

Groups	TC (mg/dl)	TG (mg/dl)	LDL (mg/dl)	HDL (mg/dl)	CRR	AC
-ve ctrl	92.7 ± 5.2	70.0 ± 4.9	62.0 ± 6.4	16.6 ± 2.0	5.64 ± 0.8	4.64 ± 0.8
Inf + CQ	83.6 ± 6.9	55.8 ± 5.5	45.3 ± 7.8	27.1 ± 2.5 ^c^	3.10 ± 0.4	2.10 ± 0.4
Inf + AI	80.1 ± 5.0 ^a^	54.1 ± 5.7 ^c^	39.1 ± 3.5 ^a^	30.1 ± 4.0 ^c^	2.68 ± 0.2 ^b^	1.68 ± 0.2 ^b^
Inf + PS	65.9 ± 4.3 ^b^	56.5 ± 5.6 ^c^	25.2 ± 7.4 ^b^	29.3 ± 3.7 ^c^	2.29 ± 0.4 ^b^	1.29 ± 0.4 ^b^
Inf + AI + PS	72.7 ± 6.6 ^b^	58.6 ± 5.1 ^c^	31.4 ± 7.1 ^b^	29.5 ± 2.6 ^c^	2.47 ± 0.3 ^b^	1.47 ± 0.3 ^b^
Baseline	70.4 ± 5.0	57.2 ± 6.6	28.1 ± 8.8	30.8 ± 4.3	2.32 ± 0.3	1.32 ± 0.3

-ve ctrl—malarous dams untreated, inf + CQ—malarous dams treated with chloroquine, inf + AI—malarous dams treated with *A. indica*, inf + PS—malarous dams treated with pear seeds, inf + AI + PS—malarous dams treated with *A. indica* and pear seeds, baseline—non malarous rats, ^a^—no significant difference (*p* < 0.05) with the chloroquine-treated malarous dams, ^b^—no significant difference with the non malarous dams, ^c^—no significant difference with both the chloroquine-treated malarous dams and non malarous dams. TC-total cholesterol, TG-triglycerides, HDL-high-density lipoprotein cholesterol, LDL-low-density lipoproteins cholesterol, CRR-cardiac risk ratio, AC-atherogenic coefficient.

## Data Availability

Data is available with reasonable request made to the corresponding author.
